# *PagERF16* of *Populus* Promotes Lateral Root Proliferation and Sensitizes to Salt Stress

**DOI:** 10.3389/fpls.2021.669143

**Published:** 2021-06-04

**Authors:** Shengji Wang, Juanjuan Huang, Xingdou Wang, Yan Fan, Qiang Liu, Youzhi Han

**Affiliations:** ^1^College of Forestry, Shanxi Agricultural University, Jinzhong, China; ^2^College of Forestry, Hebei Agricultural University, Baoding, China

**Keywords:** salt sensitivity, *PagERF16*, lateral root growth, transgenics, poplar

## Abstract

The aggravation of soil salinization limits the growth and development of plants. The AP2/ERF transcription factors (TFs) have been identified and play essential roles in plant development and stress response processes. In this study, the function of *PagERF16* was detected using the overexpressing (OX) and RNAi transgenic poplar 84K hybrids. Plant growth, stomatal conductance, antioxidant enzymes activity, and *PagERF16* co-expressed *TFs* were analyzed using morphological, physiological, and molecular methods. OX showed a more robust lateral root system with a bigger diameter and volume compared to the wild-type plants (WT). Physiological parameters indicated the bigger stomatal aperture and lower stomatal density of OX along with the lower Catalase (CAT) activity and higher malondialdehyde (MDA) content contributed to the salt sensitivity. The plant height and rooting rate of OX and RNAi were significantly worse compared to WT. Other than that, the morphology and physiology of RNAi plants were similar to WTs, suggesting that the function of *PagERF16* may be redundant with other TFs. Our results indicate that when *PagERF16* expression is either too high or too low, poplar growth and rooting is negatively affected. In addition, a downstream target TF, NAC45, involved in Auxin biosynthesis, was identified and *PagERF16* could directly bind to its promoter to negatively regulate its expression. These results shed new light on the function of ERF TFs in plant root growth and salt stress tolerance.

## Introduction

Abiotic stresses, such as extreme temperatures, drought, and soil salinity, are identified to be major adverse environmental conditions that plants often encounter (Zhu, 2016; Gong et al., 2020; Ritonga and Chen, 2020). These stressors often limit the geographic distribution of plant species, affect their growth and development, and reduce biomaterial and bioenergy production (Zhu, 2016; Li et al., 2019). For salt stress, it is important to distinguish primary stress signals from secondary signals that are caused by excess salt. The primary signal is hyperosmotic stress, while the secondary effects of salt stress include oxidative stress, damage to cellular components, and metabolic dysfunction. Although some cellular responses result from primary stress signals, others arise primarily from secondary signals (Zhu, 2002, 2016). Plants dehydrate during hyperosmotic conditions, whereupon they close their stomata to avoid further water loss, while simultaneously experiencing a stress-induced reduction in growth (Jung et al., 2017). As is well-known, roots are identified as the first organs to sense soil moisture conditions, and a robust root system is key to improve plant growth and stress tolerance under an osmotic environment (Matsuo et al., 2008; Uga et al., 2013). However, the genetic and regulatory mechanisms that confer root-mediated salt stress tolerance still remain poorly understood.

Several plant mechanisms that respond to salt stress are regulated by a subset of salt stress-responsive transcription factors (TFs) (Zhu, 2002; Chinnusamy et al., 2004; Song et al., 2016; Chen et al., 2019). Members of the AP2/ERF, MYB, bZIP, and NAC TF families are identified to play key roles in regulating plant salt tolerance mechanisms as salt stress induces or inhibits their expression; this regulation is followed by the activation of downstream salt-correlated genes that are required for plant growth and development (Nakashima et al., 2014; Lee et al., 2016). The differential expression of these *TFs* typically results from changes in the levels of specific epigenetic modifications on the genes for these TFs through stress signal transduction (Jaenisch and Bird, 2003; Kouzarides, 2007). The overexpression of salt stress-related TFs can enhance plant stress tolerance. For example, overexpression of the TF *DREB1A* in transgenic *Arabidopsis* and rice has activated the expression of many stress tolerant genes, which in turn increased plant tolerance to drought, salt, and freezing (Liu et al., 1998; Gilmour et al., 2000; Datta et al., 2012). Expression of the *BplMYB46* is seen to improve salt and osmotic tolerance in *Betula platyphylla* by affecting the expression of genes which include *SOD, POD*, and *P5CS* (Guo et al., 2017). It has been shown that a rice stress-responsive TF encoded by the rice *NAC1* gene (*SNAC1*) plays an essential role in drought stress tolerance. Plants expressing *SNAC1* have displayed significantly enhanced tolerance to drought and salinity in multiple generations, and they were found to contain higher levels of water and chlorophyll in their leaves, compared to the wild types (Saad et al., 2013). *NAC45* from poplar is induced by salt stress and hypersensitizes to salt stress (Zhang et al., 2015). Moreover, *ATAF2* as the homologous gene of *NAC45* negatively regulates plant abiotic stress defense and is involved in the auxin biosynthesis pathways by inducing the expression of *NIT2* (Delessert et al., 2005; Huh et al., 2012; Wang and Culver, 2012; Zhang et al., 2015).

Members of the AP2/ERF family, which contain the conserved AP2 domain, are especially spread in plants and are classified into ten subgroups (Nakano et al., 2006). AP2/ERF TFs regulating plant growth, root development, and stress tolerance have been revealed. In *Arabidopsis thaliana, PUCHI*, which encodes a AP2/EREBP TF, contributes to lateral root formation and morphogenesis (Hirota et al., 2007). Overexpression of the *A. thaliana HARDY* gene improved rice water use efficiency with enhanced root strength, branching, and cortical cells under drought and salt conditions (Karaba et al., 2007). Both the *ERF3* and *AP37* rice genes play positive roles in promoting crown root development, and the overexpressed plants display significant grain yield increase under the previously recorded drought conditions (Oh et al., 2009; Zhao et al., 2015). Additionally, the overexpression of *OsERF71* and *OsERF137* provides drought resistance and increased grain yield by altering the root structure of rice (Ambavaram et al., 2014; Lee et al., 2016). In *Populus, PtaERF003* was determined to have a positive effect on both adventitious and lateral root proliferation (Trupiano et al., 2013).

In this study, we detected the function of *PagERF16*, an ERF TF gene, with molecular biology and physiology indexes using overexpressing (OX) and RNAi transgenic poplar 84K hybrids (*Populus alba* × *P. glandulosa*). OX plants displayed salt hypersensitivity characteristics and a robust lateral root system compared to wild-type (WT) plants. By cross-referencing RNA-seq data, a putative target*, NAC45*, was identified. Yeast one hybrid assay and RT-qPCR indicated that *PagERF16* negatively regulated the expression of *NAC45* by binding to the DRE motif in its promoter. This will provide a theoretical basis for the study of ERF TFs in poplar growth and salt sensitivity.

## Materials and Methods

### Plant Materials and Growth Conditions

Poplar 84K was used for all experiments. To examine the spatiotemporal expression of *PagERF16*, 1-month-old clonally propagated seedlings grown in a chamber (21–25°C, 16-h light/8-h dark cycle with supplemental light of ~300 μEm^−2^s^−1^, three-band linear fluorescent lamp T5 28W 6400K, and 60–80 % humidity) with 1/2 Murashige and Skoog (MS) plant medium were treated with 100 mM NaCl solution for 24 and 48 h. The roots, stems, mature leaves (second), and shoots were excised from the plants, immediately frozen in liquid nitrogen, and stored at −80°C until use (Li et al., 2012; Lin et al., 2013; He et al., 2019).

### Total RNA Extraction

Total RNA from the collected poplar 84K materials was extracted using a RNAprep Pure Plant Kit (TIANGEN, Beijing, China) as previously described (Yao et al., 2016; Li et al., 2019). RNA quality and quantity were measured using a Bio-Spectrometer fluorescence photometer (Eppendorf, Hauppauge, NY, USA). The extracted RNA was then used for RT-qPCR, gene cloning, and RNA-seq.

### RT-qPCR Analysis

RT-qPCR was performed as previously described, and cDNAs were synthesized using a Fast Quant RT Kit (TIANGEN), according to the manufacturer's instructions (Wang et al., 2014; Yao et al., 2016). RT-qPCR was performed using TB Green Premix Ex Taq ^TM^ II (TaKaRa, Dalian, China) on an Agilent Mx3000P Real-Time PCR System (Li et al., 2012, 2019). The primers, developed using Primer Premier v6.0 software (PREMIER Biosoft, Palo Alto, CA, USA), were listed in Supplementary Table 1 (He et al., 2019). *Actin* and *EF1* was used as the housekeeping reference gene. PCR amplification in the logarithmic phase for each DNA sample was analyzed (Li et al., 2019).

### Generation of Transgenic Poplar

The coding region of *PagERF16* was amplified from poplar 84K and inserted into a pART-CAM vector under the control of the cauliflower mosaic virus (CaMV) 35S promoter at the XhoI and XbaI sites after sequence confirmation to generate an overexpression (OX) construct (Gang et al., 2019; He et al., 2019). RNAi construct was designed for the downregulation of *PagERF16*. Specific sequence of *PagERF16* were amplified using primers *PagERF16-RNAi_1* and cloned into pKANNIBAL vector at the XhoI and XbaI sites to form RNAi transgene fragments. After sequencing, the RNAi transgene fragments were subcloned into pRAT27 vector with primes *PagERF16-RNAi_2* at the XhoI and XbaI sites to obtain RNAi construct (Li et al., 2019). OX and RNAi constructs were then introduced into an *Agrobacterium tumefaciens* strain *GV3101* for poplar 84K transformation as previously described (He et al., 2018). The expression of *PagERF16* in transgenic (OX and RNAi) and WT plants was determined using PCR and RT-qPCR of leaf tissues as described above (He et al., 2019; Li et al., 2019). The primers used for vector construction, PCR, and RT-qPCR were summarized in Supplementary Table 1.

### Phylogenetic Analysis

Multiple sequence alignments were performed using Clustal X1.83 as previously described (Wang et al., 2019). An unrooted phylogenetic tree was constructed using MEGA 7.0.21 with the neighbor-joining method and 1,000 bootstrap replicates (Kumar et al., 2016).

### Morphological and Physiological Measurements

To confirm salt stress tolerance, shoots cut from 1-month-old OX, RNAi, and WT plants were sub-cultured on 1/2 MS medium containing 50 mM NaCl for 30 d (Yao et al., 2016). In each experiment, six plants per transgenic line and six WT plants were used. Three OX or RNAi lines served as three biological repeats, respectively. Mature leaf (second) and root images were taken using an Epson Expression 10,000 XL desktop scanner and further analyzed using the WinRHIZO system (Regent Instruments, Quebec, Canada) to obtain measurements such as the average root diameter, tips, surface area, volume, total length, leaf area, and aspect ratio (Liu et al., 2015, 2019). The color of leaf was detected using the WR SERIES COLORIMETER (FRU, Shenzhen, China) with the LAB methods according to the manufacturer's instruction.

The leaf (second) relative water content (RWC) was calculated as (FW-DW)/(TW-DW) ×100. The leaf was detached and weighted to obtain the fresh weight (FW). The leaf was placed in water for 24 h and was weighted to get the turgid weight (TW). The leaf was dried to a constant weight at 65°C and was used as the dry weight (DW) (Wang et al., 2016).

Peroxidase (POD), superoxide dismutase (SOD), Catalase (CAT) activity, malondialdehyde (MDA) content, and relative electrical conductance was measured as previously described (Yao et al., 2016; Wang et al., 2017; He et al., 2018).

Stomatal density and size were detected with leaves floated on stomatal opening buffer (containing 10 mM CaCl2, 50 mM KCl, and 5 mM MES, pH 6.15) for 3 h in the light to preopen stomata. Lower epidermal strips were collected for measurement of stomatal apertures using an Olympus BH-2 light microscope (Wang et al., 2020).

### RNA-Seq Analysis

Leaves (second) from 1-month-old OX and WT plants were used for RNA-seq. WT plants were treated with 100 mM NaCl solution (WT_S) for 24 h. A total of nine libraries, three independent transgenic lines (OX-12, OX-16, and OX-19) under normal growth conditions (materials from six plants pooled per line), three samples from the WT, and three samples from WT_S (materials pooled from six plants for each) were sequenced using IIlumina Novaseq 6,000 with the 2 ×150 bp paired reads by Majorbio (Majorbio Bio-pharm Technology, Shanghai, China). Raw data about 4.0 GB per sample was obtained and clean reads were mapped to the *P. trichocarpa* genome v.4.0 using TopHat2 (Kim et al., 2013). Gene expression was reported as transcripts per million reads (TPM). Differentially expressed genes (DEGs) between OX and WT, as well as between WT and WT_S, were identified using DESeq2 with a log_2_fold-change (|log_2_FC|) ≥1 and an adjusted *P*-value <0.05 as cutoffs (Love et al., 2014; Schurch et al., 2016). The data were analyzed on the free online platform Majorbio Cloud (www.majorbio.com).

### Motif Discovery

Conserved motifs of proteins were detected using the program MEME version 5.0.2 (Bailey et al., 2009). MEME was run with the following parameters: any number of repetitions, a maximum number of 6 motifs, and between 6 and 50 residues for optimum motif widths. Promoter sequences (2 kb upstream of the translation start site) were blasted and obtained from the Phytozome v12.1 database. The cis-elements prediction and location in promoters were performed using the PlantCARE (http://bioinformatics.psb.ugent.be/webtools/plantcare/html/).

### Yeast One-Hybrid Assays

The yeast one-hybrid assay was performed to verify the physical interactions between *PagERF16* and the promoter of downstream target genes (Luo et al., 2020). The promoter sequence (−525 bp to −197 bp upstream of the translation start site) of *NAC45* containing DRE cis-element was amplified using primers *Promoter-NAC45* from 84K poplar and cloned into the pAbAi vector. The CDS of *PagERF16* were cloned using primers *AD-PagERF16* and inserted into the pGADT7 vector (Clontech, CA, USA). The primers used were listed in Supplementary Table 1. The *pGADT7-Rec-53* and *p53-AbAi* were used as positive control and *pGADT7-ERF16* and *pAbAi* served as the negative control. The recombinant plasmids were transformed into yeast *Y1H* Gold strains and plated on the SD/-Leu medium containing either 0 or 200 ng/ml Aureobasidin A (AbA).

### Statistical Analysis

One-way analysis of variance was used to detect the significance of the differences among morphology and physiology indexes using OriginPro 2016 (Northampton, MA, USA). A Tukey test was performed to determine significant differences by a *p*-value cutoff value of 0.05. *t*-test was used to analyze gene expression differences between transgenic and WT plants.

## Results

### *PagERF16* Is a Salt Stress Related Transcription Activator

**Figure 1:**
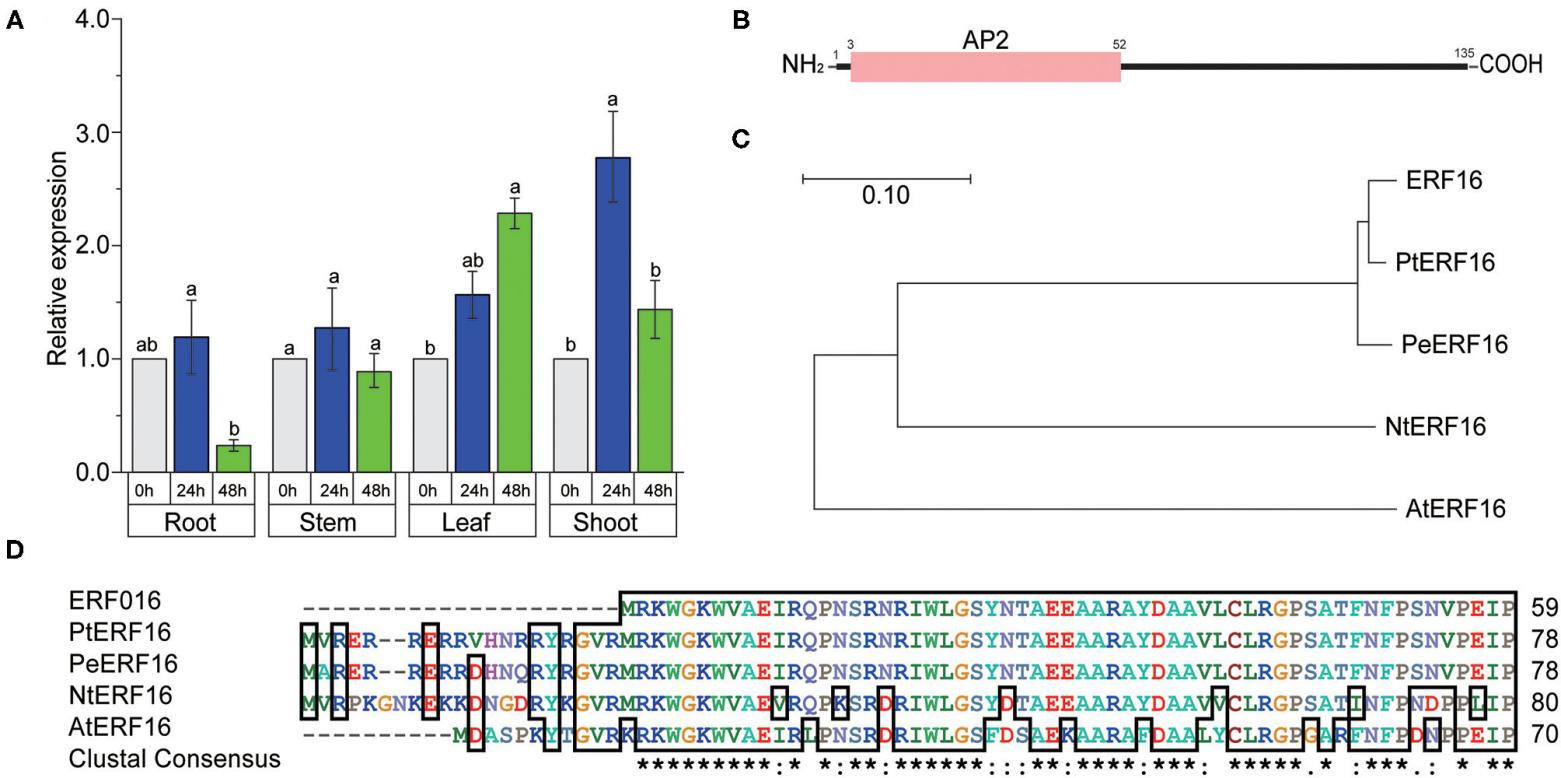
Spatio-temporal expression pattern and sequence alignment of *PagERF16*. **(A)** Relative expression of *PagERF16* in roots, stems, leaves, and shoots, respectively, under 100 mM NaCl. Different lowercases above the bar chart indicate significant differences among samples (*p* ≤ 0.05). **(B)** AP2 conserved domain architecture of PagERF16 of poplar. **(C)** Phylogenetic analysis of PagERF16 homologs in *P. trichocarpa, P. euphratica, Nicotianu tabacum*, and *Arabidopsis thaliana*. **(D)** Multiple sequence alignment of PagERF16 amino acid sequence of *Populus trichocarpa, P. euphratica, Nicotianu tabacum*, and *Arabidopsis thaliana*.

The study has indicated that 25 *ERF* TF genes, including *ERF16*, were up- or downregulated when *Populus* was exposed to NaCl, KCl, CdCl_2_, and PEG 6,000 solutions (Yao et al., 2019). To elucidate the potential function of *PagERF16* in poplar 84K salt tolerance and growth, the spatiotemporal expression pattern was analyzed using RT-qPCR with primers *PagERF16-RT* under 100 mM NaCl treatment (Supplementary Table 1). When subjected to salt stress, *PagERF16* in roots was determined to be sensitive to salt stress, whose transcript levels decreased to significantly lower level at 48 h (Figure 1A). However, expression of *PagERF16* in shoots and leaves was found to be highly induced at 24 or 48 h, respectively (Figure 1A). On the other hand, the expression of *PagERF16* in stems showed no significant change. These results suggested that *PagERF16* may function in roots development and be suppressed by salt stress at least in roots.

To detect the feasible molecular structure of *PagERF16*, the cDNA was cloned from poplar 84K using PCR with *PagERF16-CAM* primers (Supplementary Table 1). The coding sequence (CDS) of *PagERF16* was 408 bp encoding 106 amino acid residues. Motif discovery analysis showed that *PagERF16* has a conserved AP2 domain located in amino acid sequences 3–52 (Figure 1B). A phylogenetic tree with homologous proteins from other plant species was constructed, and *PagERF16* was roughly homologous with *NtERF16* of *Nicotiana tabacum* and *AtERF16* of *Arabidopsis thaliana* (Figure 1C). Multiple alignment of the amino acid sequences indicated that *PagERF16* shared common conserved domains with *PtERF16* of *P. trichocarpa* and *PeERF16* of *P. euphratica* (Figure 1D).

### *PagERF16* Overexpressing Poplar Is Sensitive to Salt Stress

To investigate the biological functions of *PagERF16*, transgenic poplar 84K overexpressing (OX) or downregulating (RNAi) *PagERF16* were generated under the control of a CaMV 35S promoter. The transgenic lines were verified using PCR with *PagERF16-T1* primers, which were composed of a forward primer from the promoter of CaMV 35S and a reverse primer from *PagERF16* (Supplementary Table 1; Supplementary Figure 1A). In total, 19 OX and nine RNAi transgenic lines were obtained in this study (Supplementary Figures 1A,B). RT-qPCR using the leaf tissues was used to cross-verify the transgenic plants at a transcript level. Three OX and three RNAi transgenic lines (OX-12, OX-16, OX-19, RNAi-1, RNAi-4, and RNAi-5) with the highest and lowest *PagERF16* transcript level were selected for further analysis (Supplementary Figures 1C,D). The expression of *PagERF16* in selected OX was significantly higher than WT while that in RNAi was only 0.6 times to WT. The indistinctive expression between the RNAi and WT may be one of the reasons contributing to the phenotypic similarity between RNAi and WT.

**Figure 2:**
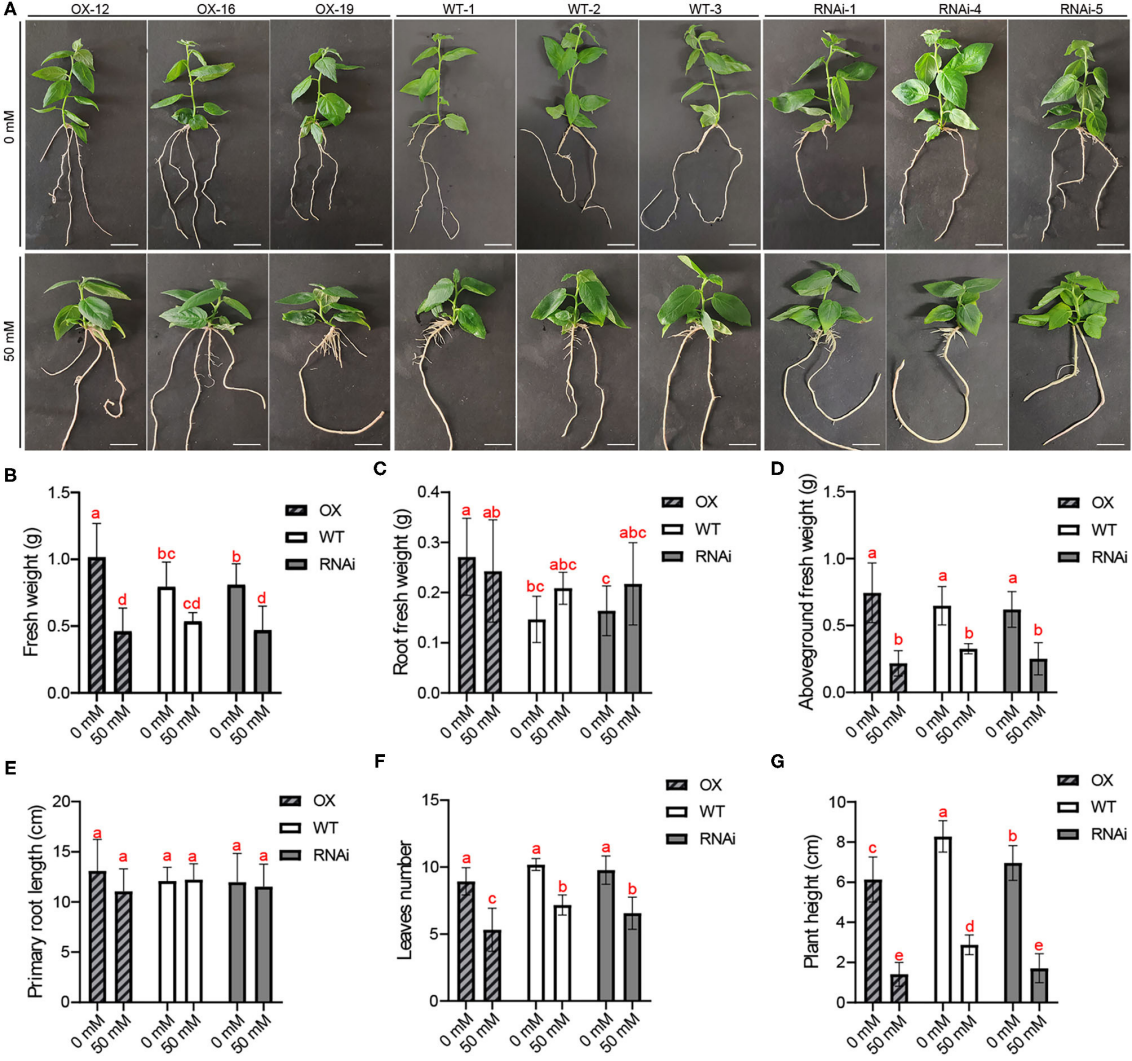
PagERF16 was sensitive to salt stress. **(A)** Morphology of the OX, RNAi, and WT grown on medium containing 50 mM NaCl for 30 d. Bars, 2 cm. **(B)** Fresh weight of whole plant. **(C)** Root fresh weight. **(D)** Fresh weight of aboveground tissues. **(E)** Length of primary root. **(F)** Numbers of leaves per plant. **(G)** Height of plant. The plot represents the mean ± SD of six plants per line. Three OX and RNAi lines serve as three biological repeats, respectively. Different lowercases indicate significant differences among samples (*p* ≤ 0.05).

Under normal growth condition (NC, 0 mM NaCl), the plant height of OX was shorter than that of WT and RNAi (Figures 2A,G). However, an obvious difference was found that the lateral roots of OX were thicker and stronger compared to the WT and RNAi (Figure 2A). The root fresh weight of OX was much bigger than that of WT and RNAi while the primary root length was similar (Figures 2C,E). The fresh weight of the aboveground tissues among the OX, RNAi, and WT had no significant differences (Figure 2D). These results indicated that the robust lateral roots of OX made a major contribution to the bigger fresh weight of OX (Figure 2B). The numbers of leaves per plant was similar among the different genotypes (Figure 2F). The morphology of RNAi plants was relatively similar to the WT, although WT plants were higher (Figure 2). In addition, we found that the leaf color was different among OX, RNAi, and WT, but the difference could not be well-distinguished in a picture (Figure 2A). We quantify the color of leaf using LAB method in the following experiments.

When sub-cultured under salt stress (50 mM NaCl) conditions for 30 d, the plant height of transgenic and WT plants decreased but the roots thickened to different degrees (Figure 2). The fresh weight and aboveground fresh weight were similar among OX, RNAi, and WT, but the reduction of OX compared to that under normal condition was biggest (Figures 2B,D). The root fresh weight of OX was relatively reduced to that under normal condition while WT and RNAi were increased (Figure 2C). We also found that the salt stress did not significantly inhibit the elongation of primary roots but decreased the number of leaves, especially in OX (Figures 2E,F). Above all, the overexpression of *PagERF16* made poplar sensitive to salt stress.

### *PagERF16* Enhances Lateral Root Growth

**Figure 3:**
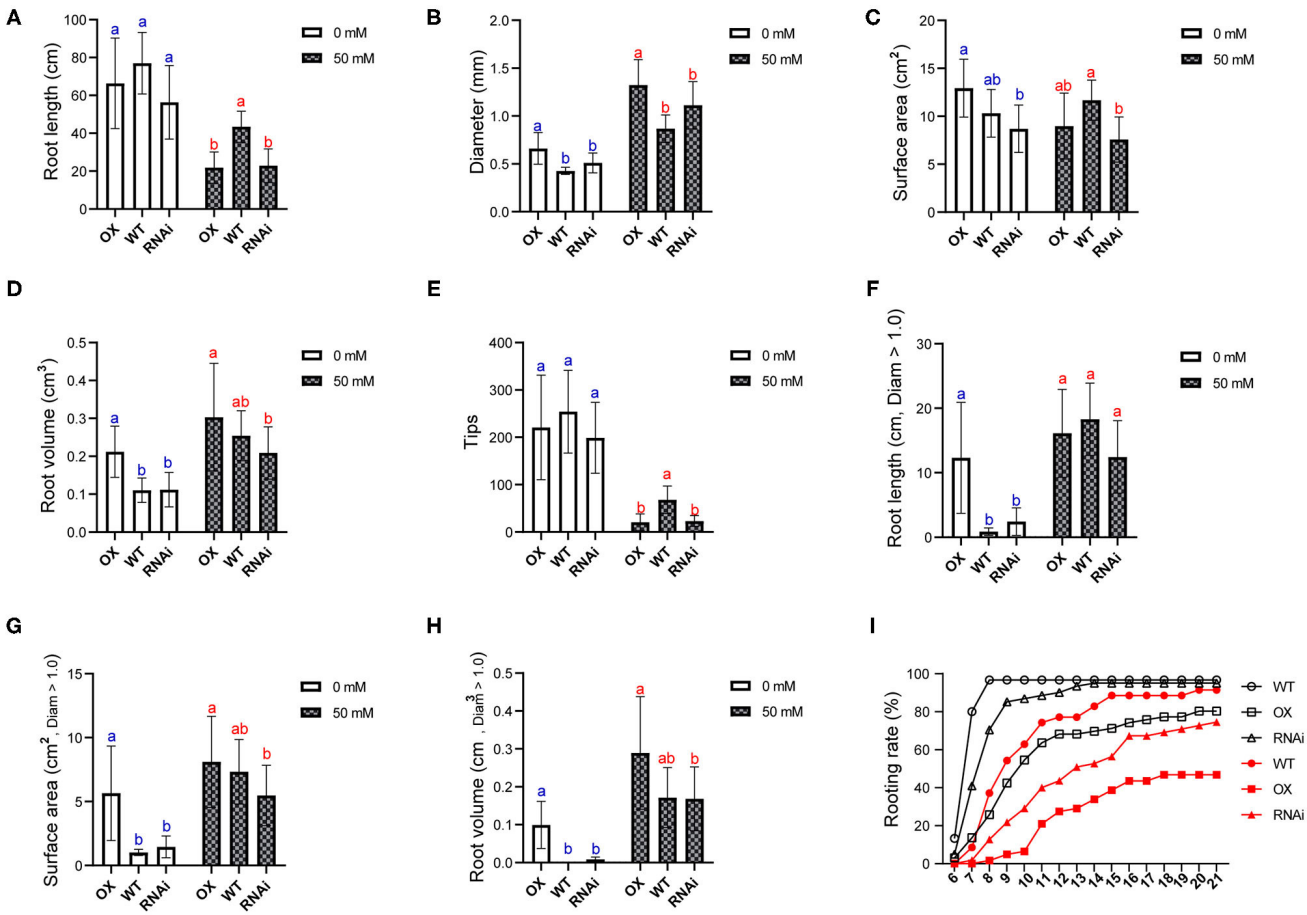
PagERF16 promoted lateral root proliferation by increasing the diameter and volume. **(A)** Total length of root. **(B)** Average diameter of root. **(C)** Total surface area of root. **(D)** Total volume of root. **(E)** Tips of root system. **(F–H)** Represent the length, surface area, and volume of roots with diameter >1.0 mm, respectively. The plot represents the mean ± SD of six plants per line. Three OX or RNAi lines serve as three biological repeats. Different lowercases indicate significant differences among genotypes under the control (blue) or salt stress (red) conditions (*p* ≤ 0.05). **(I)** Rooting rate of 60 shoots per genotype (OX, RNAi, and WT) sub-cultured on medium without (black) or with 50 mM NaCl (red).

To verify the effect of *PagERF16* on the lateral root system, the roots of OX, RNAi, and NT were scanned and analyzed using WinRHIZO software. Under normal condition, total root length and tips showed no significant difference among OX, RNAi, and WT (Figures 3A,E), but the root average diameter and volume of OX was much bigger than those of WT and RNAi (Figures 3B,D). The surface area of OX was significantly bigger than that of RNAi, although both showed no difference to WT (Figure 3C). Individual statistical analysis of roots with Diameter > 1.0 was made and it was found that the length, surface area, and volume of OX were significantly bigger than those of WT and RNAi (Figures 3F–H). All above parameters of RNAi were similar to those of WT.

When exposed to salt stress, the total root length and tips of all genotypes were reduced but diameter, surface area, and volume were increased (Figure 3). The root length and tips of OX and RNAi were smaller than those of WT, indicating that salt stress inhibited OX and RNAi lateral root elongation and numbers to a much deeper degree (Figures 3A,E). The root length and tips of RNAi was similar to those of OX. The diameter and volume of OX were bigger than those of RNAi, suggesting that although the salt stress increased the diameter and volume of RNAi, the diameter increment caused by the expression of *PagERF16* to OX still cannot be ignored (Figures 3B,D). The diameter and volume of RNAi were similar to those of WT while surface area was smaller. Parameters of roots with Diameter > 1.0 were not significantly different between WT and OX or WT and RNAi, but those of OX were much bigger than those of RNAi (Figures 3F–H).

In addition, the rooting rate of sub-cultured shoots were monitored. Under normal condition, the initial rooting time and rooting rate of OX was worse than that of WT, while RNAi only showed a difference from WT in rooting time. Nevertheless, the rooting rate and rooting time of WT were much better than those of OX and RNAi after being treated with salt stress, especially OX. Overexpressing of *PagERF16* significantly reduced the rooting rate of poplar and postponed rooting time, which may be the reason that primary root length of OX was not elongated compared to WT (Figure 3I). All of these findings indicated that *PagERF16* functioned in poplar root growth, especially in lateral root proliferation.

### PagERF16 Reduces Stomatal Density and Increases Stomatal Width

**Figure 4:**
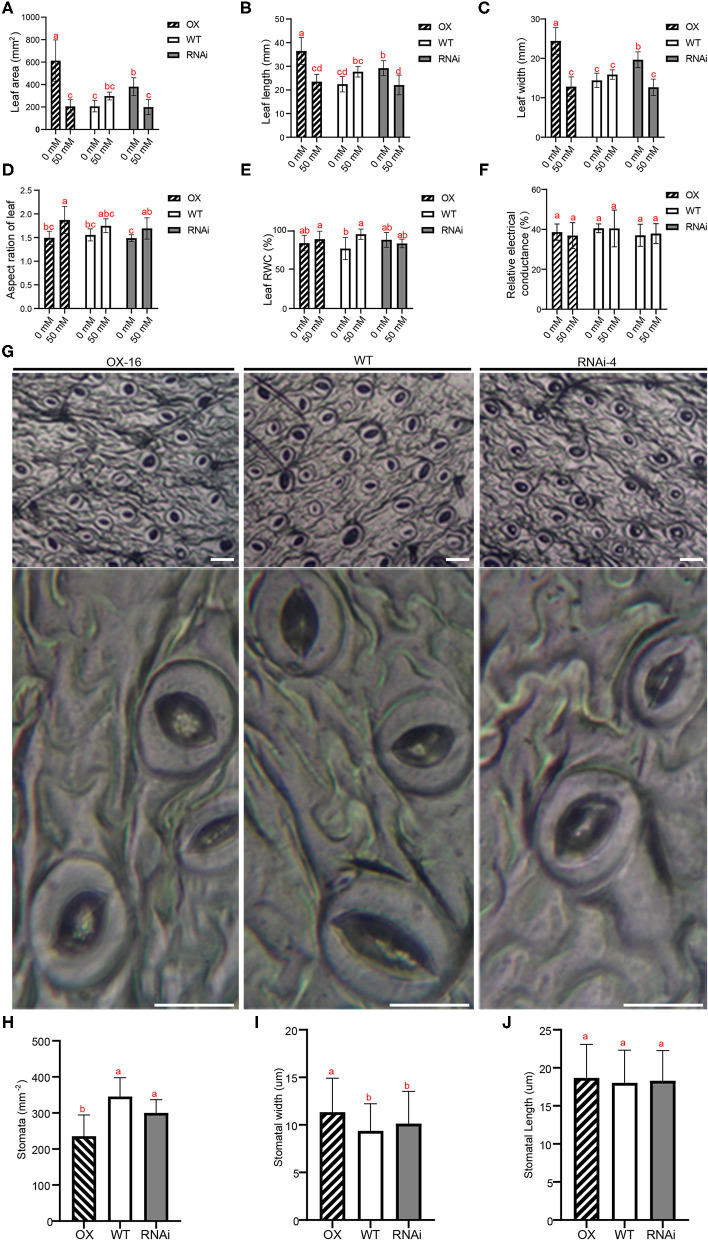
PagERF16 decreased stomatal density and increased stomatal width. **(A–D)** Represent area, length, width, and aspect ratio of the second mature leaf. **(E)** Relative water content of the second mature leaf. **(F)** Relative electrical conductance of the second leaf. **(G)** Scanning electron micrograph of the abaxial leaf epidermis. Bars, 20 μm. **(H–J)** show the stomatal density, width, and length. The plot represents the mean ± SD of six plants per line. Three OX or RNAi lines serve as three biological repeats. Different lowercases indicate significant differences among samples (*p* ≤ 0.05).

Under normal growth conditions, the plant height of OX was smaller than that of WT (Figure 2G), but the parameters of leaf, including leaf area, length, and width, were bigger (Figures 4A–C). RNAi showed similar leaf phenotype with OX that was bigger than WT. The leaf aspect ratio of these three genotypes was not different (Figure 4D). These results indicated that *PagERF16* could affect the size of leaves but not the shape characteristics. After being treated with salt stress, the leaf size of OX and RNAi was significantly decreased, especially the OX, while WT showed no change (Figures 4A–C). The leaf aspect ratio of these three genotypes increased to different degrees, suggesting that salt stress induced the leaf shape to become slenderer (Figure 4D). In addition, the leaf color of OX was more yellowish green compared to WT and the difference decreased after being treated with salt stress (Supplementary Table 2).

To investigate whether changes of leaf phenotype contributed to salt stress tolerance of poplar, the leaf RWC and electrical conductance were measured. Results showed that both of these two indexes were not significantly changed among the three genotypes with/without salt stress treatment (Figures 4E,F). What caught our attention was that the stomatal density and size was different among transgenic plants and WT (Figures 4G–J). The stomatal density of OX decreased but the width increased compared to the WT. However, the leaf characteristics of RNAi were similar to WT.

### *PagERF16* Inhibits Antioxidant Enzymes Synthesis

**Figure 5:**
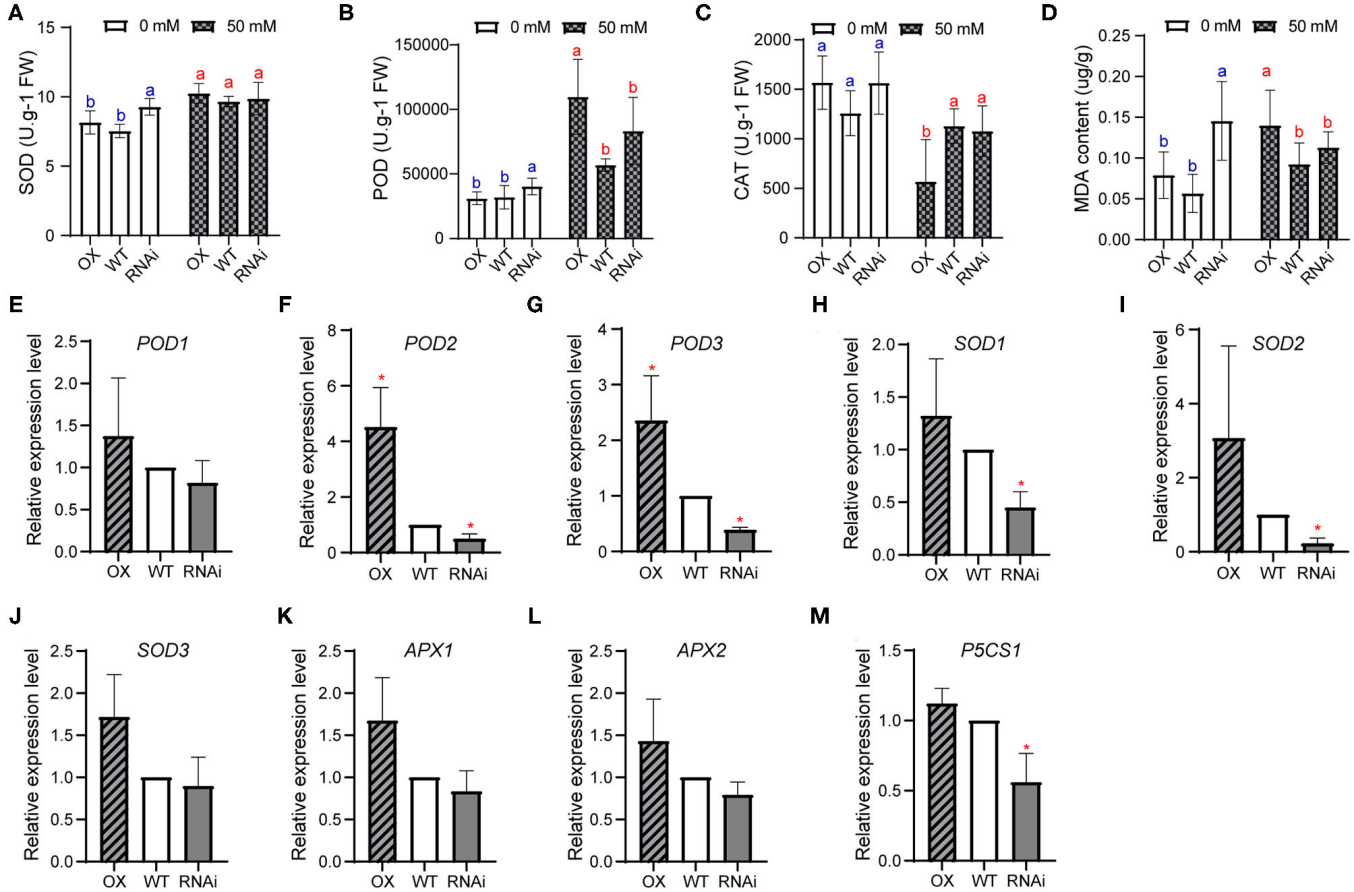
PagERF16 modulated the physiology and gene expression relevant to antioxidant enzymes. **(A–D)** Superoxide dismutase (SOD), peroxidase (POD), catalase (CAT), and MDA contents. The plot represents the mean ± SD of six plants per line. Three OX or RNAi lines serve as three biological repeats. Different lowercases indicate significant differences among samples (*p* ≤ 0.05). **(E–M)** Expression of *POD1, POD2, POD3, SOD1, SOD2, SOD3, APX1, APX2*, and *P5CS1* in transgenic and WT plants. The plot represents the mean ± SD of three repeats. Asterisks denote significant differences between transgenic and WT: *p ≤ 0.05.

When plants encounter a severe environment, antioxidant enzymes will increase to reduce the contents of reactive oxygen species (ROS). ROS accumulation is an important messenger in stomatal movement (opening and closure), leading to reduced water loss (Zhang et al., 2020). To detect the function of *PagERF16* in stomatal movement and ROS accumulation, we analyzed the activity of antioxidant enzymes. Under normal conditions, the POD and SOD activities and MDA content of OX were similar to those of WT but significantly lower than those of RNAi (Figures 5A,B,D). The CAT activity of these three genotypes showed no difference (Figure 5C). These indicated that repressing *PagERF16* increased the antioxidant enzymes synthesis of poplar. When treated with salt stress, POD and SOD activities and MDA content were all induced and OX had more POD and MDA than WT and RNAi. The CAT activity was inhibited by salt stress.

The transcript level of genes relevant to ROS accumulation was also explored in this study. Results showed that the OX plants had relatively higher expression levels in all of these nine genes (Figures 5E–M). Expression of *POD2* and *POD3* were significantly induced in OX compared to WT while suppressed in RNAi (Figures 5F,G). Moreover, *SOD1, SOD2*, and *P5CS1* were downregulated in RNAi (Figures 5H,I,M). Expression of *POD1, SOD3, APX1*, and *APX2* were not different from each other among the three genotypes (Figures 5E,J–L). These indicated that overexpressing *PagERF16* could up-regulate genes involved in POD metabolism process to modulate poplar salt stress tolerance, while repressing *PagERF16* inhibited the synthesis of POD, SOD, and P5CS1 proteins.

### *PagERF16* Negatively Regulates the Expression of *NAC45*

**Figure 6:**
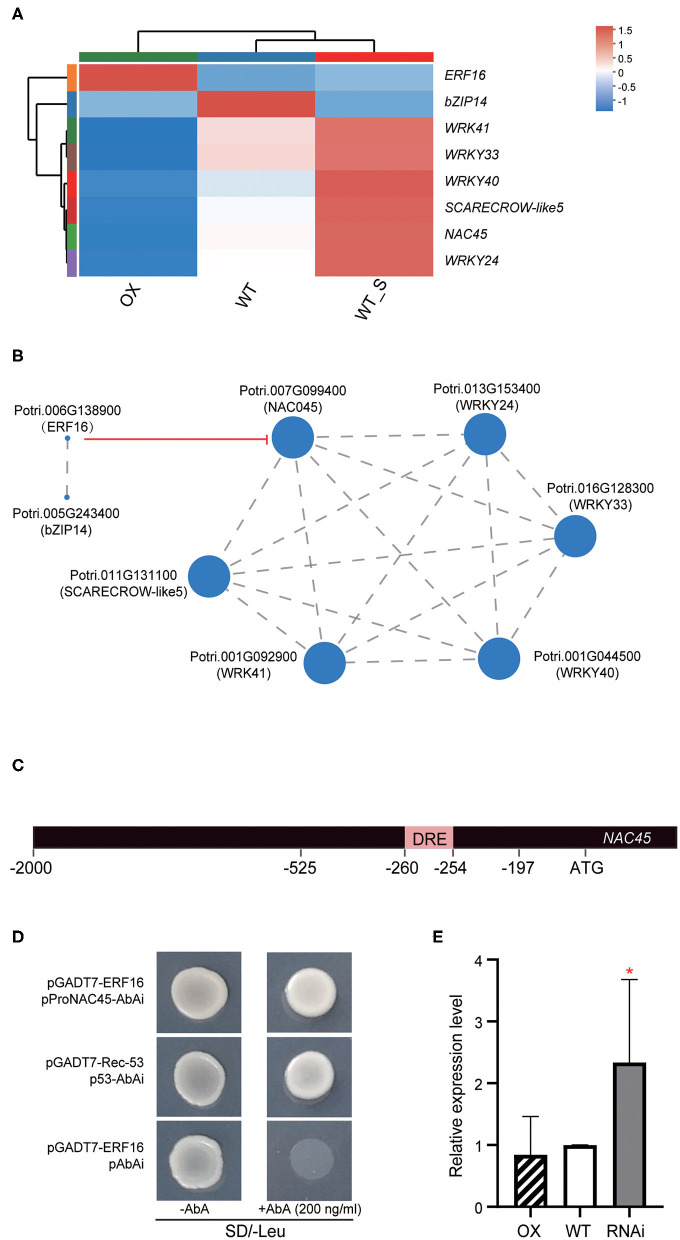
PagERF16 negatively regulated the expression of NAC45. **(A)** Expression pattern of differentially expressed TFs related to salt stress and PagERF16. WT_S, WT treated with 100 NaCl for 48 h. Red denotes high expression and blue indicates low expression. **(B)** Correlation analysis of co-expressed TFs using Spearman methods. Each node (Node) in the Figure represents a gene, and the wiring between nodes represents the correlation of gene expression. The larger the node, the more the number of expression correlations between this gene and other genes. Red line indicates PagERF16 negatively regulated the expression of NAC45. **(C)** Promoter structure of *NAC45*. **(D)** *PagERF16* directedly binding to the promoter of *NAC45* revealed by yeast one hybrid assay. The pGADT7-Rec-53 and p53-AbAi were used as positive control and pGADT7-ERF16 and pAbAi served as the negative control. The yeast Y1H Gold strains and plated on the SD/-Leu medium containing either 0 or 200 ng/ml AbA. **(E)** Relative expression level of *NAC45* in OX, RNAi, and WT. The plot represents the mean ± SD of three repeats. Asterisks denote significant differences between transgenic and WT: *p ≤ 0.05.

To unravel the regulatory function of *PagERF16*, leaf transcriptomes were analyzed in WT and OX plants using RNA-seq. DEGs in WT_S vs WT and in OX vs WT were selected using |log_2_FC| ≥ 1 and adjusted *p*-value ≤ 0.05 cutoffs (Supplementary Tables 3, 4). In total, 1,175 DEGs were identified for WT_S with 1.9 times more up- than downregulated genes (766 and 409, respectively, Supplementary Figure 2A). A much smaller amount of DEGs were identified for OX, which showed a converse distribution with 3.6 times more down- than upregulated genes (226 and 62, respectively). The TFs of the DEGs were identified using PlantTFDB 4.0 with Hmmscan (E-value <1.0E-5) methods and were annotated using BLAST (E-value < 1.0E-5). The TFs for WT_S mainly belonged to NAC, ERF, MYB-related, and WRKY TF families (Supplementary Figure 2B), whereas TFs for OX were enriched in WRKY and MYB-related TF families (Supplementary Figure 2C). In total, seven common TFs were identified for WT_S and OX plants, more than half of which (WRKY24, WRKY33, WRKY40, and WRKY41) belonged to WRKY family in prediction. There was also one NAC (NAC45), one bZIP TF (bZIP14), and one GRAS (SCARECROW-like5, Supplementary Table 5). Expression pattern showed that all of the genes were down-regulated in OX (Figure 6A). To identify target TFs that may be related to *ERF16*, a correlation analysis was performed, using Spearman method. Only *bZIP14* was directly correlated with *PagERF16* and the remaining five TFs related to each other filled in another network (Figure 6B).

To reveal the regulation relationship between *PagERF16* and the five-TFs network, *cis-*elements in the promoter regions of the inferred targets were detected using the PlantCARE programme. We found that *NAC45* contained a DRE motif which could be bound to PagERF16 (Cheng et al., 2019). Yeast one-hybrid assays showed that PagERF16 could directly bind to the promoter (−525 to −197 bp upstream of the translation start site) of *NAC45* containing the DRE motif (Figures 6C,D). In addition, RT-qPCR cross-validated that *NAC45* was highly induced in RNAi plants (Figure 6E). However, the expression of *NAC45* in OX showed no significant difference to WT. These suggested that repressing *PagERF16* could induce the expression of *NAC45*, but *PagERF16* overexpression had an insignificant effect on *NAC45* (Figures 6A,E).

## Discussion

### *PagERF16* Is a Salt-Stress-Related Transcription Activator

AtERF16 belongs to subgroup II of the ERF TF family, members of which contain CMII-1, CMII-2, and CMII-3, the three conserved motifs in the C-terminal region adjacent to the AP2/ERF domain (Nakano et al., 2006). ERF in this subfamily most likely acts as a transcriptional activator through binding to the GCC-box or DRE (dehydration responsive element) promoter element and may be involved in the regulation of gene expression by biotic or abiotic stress factors and by components of stress signal transduction pathways. Expression of *AtERF014* was determined to be induced by *Pseudomonas syringae* pv. *tomato* (*Pst*) and *Botrytis cinerea* (*Bc*). *AtERF014*-overexpressing plants displayed increased *Pst* resistance but decreased *Bc* resistance, whereas *AtERF014*-RNAi plants exhibited decreased *Pst* resistance but increased *Bc* resistance (Zhang et al., 2016). Additionally, ERFs in subgroup II were also involved in plant secondary metabolism and the growth/development process. AtERF19 plays a primary role in plant growth and development and causes an increased tolerance to water deprivation, strengthening their chances of reproductive success (Scarpeci et al., 2017). Previous studies suggest that *ERF16* is located in the nucleus of the poplar cell and could specifically bind to the DRE motif to regulate abiotic stress of transgenic plants (Cheng et al., 2019). However, the function and molecular mechanism of *ERF16* in poplar root growth and salt stress tolerance remain to be clarified. In order to evaluate the function of *PagERF16* in ligneous plants, the gene was cloned from 84K poplar and was roughly homologous with *AtERF16*, suggesting that it may play a role in poplar growth and stress tolerance (Figures 1C,D). *PagERF16* transcript levels in roots were sensitive to salt stress that decreased significantly after being exposed to NaCl but induced in leaves (Figure 1A). Similar results were obtained in previous studies, in which the spatiotemporal expression pattern of *ERF16* was reported and possible functions involved in salt stress were inferred (Cheng et al., 2019; Yao et al., 2019). This suggests that *PagERF16* may have a function in the root tissues that affects the salt sensitivity of poplars. The induced expression of *PagERF16* in leaves maybe related to the stomatal density reduction and stomatal width increase, which modulate plant adaptation to salt stress (Figures 4H,I).

### *PagERF16* Promotes Lateral Root Proliferation

Members of ERF family are involved in regulating lateral rooting. Overexpressed *PtAIL1* is able to grow more adventitious roots, whereas RNA interference mediated the downexpression of *PtAIL1* expression, which leads to a delay in adventitious root formation (Rigal et al., 2012). PtaERF003 function is linked to the auxin signal transduction pathway and has a positive effect on lateral root proliferation in poplars (Trupiano et al., 2013). In this study, 1-month-old OX plants with overexpressed *PagERF16* had robust lateral roots with bigger root diameter and fresh weight than WT (Figures 1, 2). We also found that overexpressing *PagERF16* could reduce the rooting rate of poplar and postpone rooting time, which led to no change in the length of primary root (Figure 3). All of these resulted in the lateral root proliferation and thickening of OX. Meanwhile, the repressing transgenic plants using RNAi showed similar phenotypes and physiological characteristics to the wild type suggesting that function of *PagERF1*6 may be redundant with other TFs, for example *PtERF194* (Potri.018G038100). *PagERF16* was highly homologous with *PtERF194* and both of them hit the same ortholog *AtERF016* in *Arabidopsis* (Yao et al., 2019). The plant height and rooting rate of RNAi were also significantly decreased compared to those of WT (Figures 2G, 3I) suggesting that *PagERF16* expression level must be tightly regulated, since too high or too low levels negatively affect the rooting rate.

### *PagERF16* Hypersensitizes to Salt Stress

Overexpression of *ERFs* can also negatively affect plant growth and often result in dwarf plants, which was somewhat consistent with our results (Zhang et al., 2009; Lee et al., 2016; Kudo et al., 2017; Wessels et al., 2019). The plant height of OX was shorter than WT plants, but the leaves were larger (Figures 1, 4). Under salt stress conditions, plants could close their stomata to decrease water loss from leaves. In this study, the stomatal width (aperture) of OX was bigger than that of WT, which explained why it was sensitive to salt stress to a degree (Figures 4G,I). The other factor that regulates stomatal conductance is stomatal density; regulation of stomatal density is a long-term response (Wang et al., 2016). Under stress conditions, stomatal density responses differ depending on the stress intensity and species. Reduced stomatal density due to drought stress is present in wheat and umbu trees (Quarrie and Jones, 1977; Silva et al., 2009). However, an increase in stomatal density was detected in rice during moderate drought, but there was a decrease in severe drought (Xu and Zhou, 2008). Reducing stomatal density may lead to a decrease in cumulative photosynthetic activity and increase in stress threat. We think the reduction of stomatal density decreased photosynthesis and contributed to the salt sensitivity of OX under 50 mM NaCl conditions (Figures 4G,H).

Movement of stomata is induced by many factors, including ROS, ABA, and salt stress, and plays important roles in plant abiotic stress endurance (Wang et al., 2016). Under drought conditions, ROS accumulate rapidly to control the stomatal movement as a second messenger, while antioxidant enzymes will increase to reduce the content of ROS. In addition, MDA content is related to membrane lipid peroxidation and the higher MDA content represents more membrane damage. In our present study, the POD activity of OX was similar to that of WT under normal growth conditions but was higher when exposed to salt stress (Figure 5). The MDA content showed similar trends with POD activity. However, CAT activity of OX was significantly lower than that of WT. These indicated that the lower CAT activity and higher MDA content contributed to the salt sensitivity of OX. Expression of genes relevant to ROS scavenging showed that the transcript level of *POD2* and *POD3* were higher in OX, yet could not enhance poplar salt tolerance. The decreased expression of *POD2, POD3, SOD2*, and *P5CS1* of RNAi indicated that the repression of PagERF16 could inhibit their expression, which may contribute to the decrease of plant height and rooting rate.

The fact that *PagERF16* regulated both salt stress sensitivity and lateral root proliferation suggested that the influence of *PagERF16* on these two biological processes was interlinked. The transcriptome analyses offer insight into the possible mechanisms for how *PagERF16* might modulate the lateral root systems to affect salt sensitivity of poplar plants. Combined with Yeast one-hybrid assays, we found that PagERF16 could directly bind to the promoter of *NAC45* through the DRE motif to participate in the regulation of the five-TFs network (Figures 6C,D). Studies have indicated that *NAC45* is homologous with *ATAF2* of *Arabidopsis*, which is involved in auxin biosynthesis by binding to the promoter of *NIT2* and acts as a negative regulator during the plant stress response process (Delessert et al., 2005; Huh et al., 2012; Wang and Culver, 2012; Zhang et al., 2015). The *ERF16-NAC45* interaction suggested that *PagERF16* may participate in the auxin biosynthesis pathway, but whether it promotes lateral root proliferation through the *ERF16-NAC45* interaction remains to be studied. RT-qPCR showed that *PagERF16* induced the expression of *NAC45* in RNAi plants (Figure 6E). NAC45 is a negative regulator during plant stress response process; the induced *NAC45* could make RNAi poplar sensitive to salt stress. On the contrary, *PagERF16* was sensitive to salt stress and the repressed *PagERF16* would make the RNAi poplar more tolerant to salt stress. The opposite effect of induced-*NAC45* and repressed-*PagERF16* in RNAi may be another reason why the phenotype of RNAi was mostly similar to that of WT. However, the expression of *NAC45* in OX was lightly affected by *PagERF16*, indicating that the interaction relationship between *PagERF16* and *NAC45* may not function directly in regulating salt tolerance or sensitivity of poplar.

## Data Availability Statement

The datasets presented in this study can be found in online repositories. The names of the repository/repositories and accession number(s) can be found at: https://www.ncbi.nlm.nih.gov/, PRJNA716488.

## Author Contributions

SW and YH planned and designed the research and wrote the manuscript. JH, QL, XW, and YF performed experiments and analyzed data. All authors contributed to the article and approved the submitted version.

## Conflict of Interest

The authors declare that the research was conducted in the absence of any commercial or financial relationships that could be construed as a potential conflict of interest.

## Supplementary Material

Supplementary Figure 1 The transgenic lines were verified using PCR and RT-qPCR. **(A,B)** Respectively represent overexpressed and RNAi lines verified using PCR with primers composed of a forward primer from the promoter of *CaMV 35S* and a reverse primer from *PagERF16*. M, DNA marker 2000; WT, wild-type control. **(C,D)** Shows the relative expression of *PagERF16* in OX or RNAi lines. OX-12, OX-16, and OX-19 marked with red tags and RNAi-1, RNAi-4, and RNAi-5 with blue were selected for further analysis. **(E)** Shoots were sub-cultured on medium without (0 mM) or with 50 mM NaCl. Bars, 1 cm.

Supplementary Figure 2 Identification of differentially expressed TFs coexisted between OX and WT_S. **(A)** Statistics of DEGs in OX and WT_S compared to WT plants. **(B,C)** Statistics of TF family of DEGs for WT_S and OX.

Supplementary Table 1 Primer sequences used in this study.

Supplementary Table 2 Statistic analysis of leaf color of OX, RNAi, and WT using LAB methods. 0 < ΔL, white; 0 > ΔL, black; 0 < ΔA, red; 0 > ΔA, green; 0 < ΔB, yellow; 0 > ΔB, blue. ΔE, color difference, ΔE^*^ = [(ΔL^*^)^2^ + (ΔA^*^)^2^ + (ΔB^*^)^2^]^1/2^,1 < ΔE ≤ 2, not significant; 2 < ΔE, the difference is visible.

Supplementary Table 3 DEGs in WT_S vs. WT.

Supplementary Table 4 DEGs in OX vs. WT.

Supplementary Table 5 Expression and gene annotation of TFs.
