# Supplementary figures and images for: PagERF16 of Populus Promotes Lateral Root Proliferation and Sensitizes to Salt Stress
Source: Front Plant Sci. 2021 Jun 4;12:669143. doi: 10.3389/fpls.2021.669143 (PMC8213033; doi:10.3389/fpls.2021.669143)

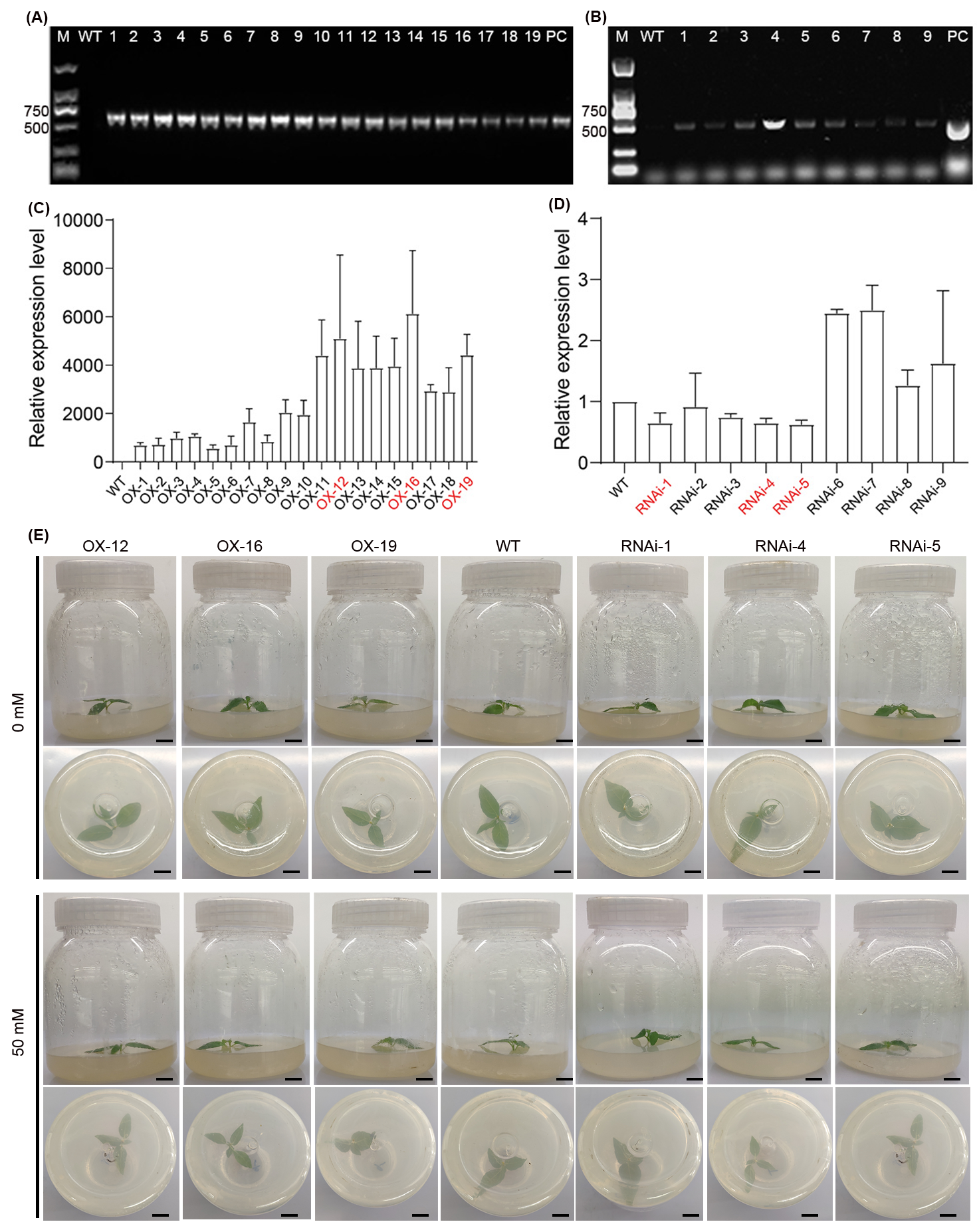

Supplement: Supplementary Figure 1 — The transgenic lines were verified using PCR and RT-qPCR. (A,B) Respectively represent overexpressed and RNAi lines verified using PCR with primers composed of a forward primer from the promoter of CaMV 35S and a reverse primer from PagERF16. M, DNA marker 2000; WT, wild-type control. (C,D) Shows the relative expression of PagERF16 in OX or RNAi lines. OX-12, OX-16, and OX-19 marked with red tags and RNAi-1, RNAi-4, and RNAi-5 with blue were selected for further analysis. (E) Shoots were sub-cultured on medium without (0 mM) or with 50 mM NaCl. Bars, 1 cm. [file Image_1.TIF]

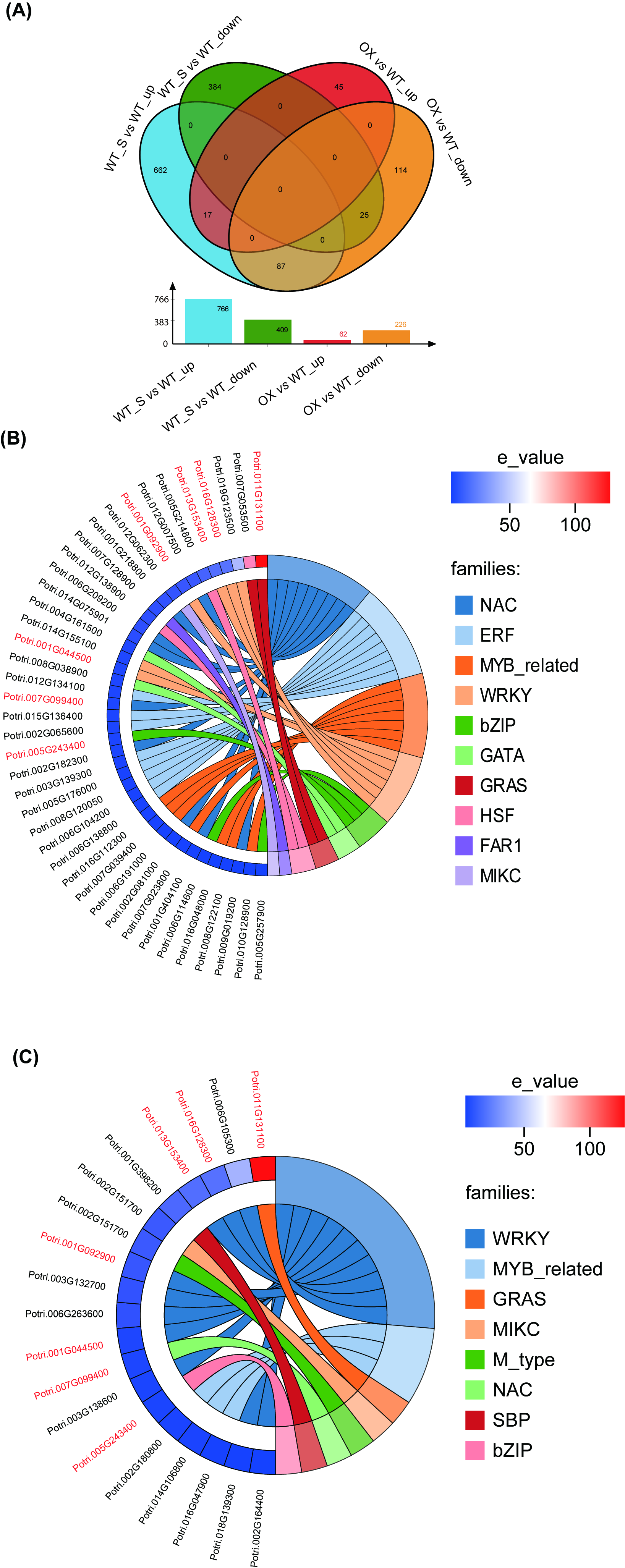

Supplement: Supplementary Figure 2 — Identification of differentially expressed TFs coexisted between OX and WT_S. (A) Statistics of DEGs in OX and WT_S compared to WT plants. (B,C) Statistics of TF family of DEGs for WT_S and OX. [file Image_2.TIF]
