# Supplementary material for: PagERF16 of Populus Promotes Lateral Root Proliferation and Sensitizes to Salt Stress
Source: Front Plant Sci. 2021 Jun 4;12:669143. doi: 10.3389/fpls.2021.669143 (PMC8213033; doi:10.3389/fpls.2021.669143)
Supplement: Supplementary Table 1 — Primer sequences used in this study. [file Table_1.DOCX]

­­Table S1 Primer sequences used for gene cloning and RT-qPCR.

| **Gene name (ID)** | ***Forward and reverse primers (5’-3’)*** | |
| --- | --- | --- |
| *PagERF16-CAM* | GGAGAGGACACGCTCGAGATGAGAAAGTGGGGAAAATGGGT | TTAAAGCAGGACTCTAGATTAAACTGTCCACACACCAGATGT |
| *PagERF16-T1* | TGACGCACAATCCCACTATC | AACTGTCCACACACCAGATGTTTG |
| *AD-PagERF16* | ATGGCCATGGAGGCCAGTGAATTCATGAGAAAGTGGGGAAAATG | CTGCAGCTCGAGCTCGATGGATCCTTAAACTGTCCACACACCAG |
| *Promoter-NAC45* | ATTGAAAAGCTTGAATTCGAGCTCCCCGACGTGGGCTCTCTTG | TACATACAGAGCACATGCCTCGAGGCCACGCGTTCCTGCTTATG |
| *PagERF16-RT* | CCGCAGAGGAAGCAGCAAGAGC | GATTCCACAATCCTCTCTGCAG |
| *NAC45-RT* | GGAACCGGGTATTGGAAAGCAAC | GCTTCTCTACGCTTCCTTTCTTG |
| *SOD1*  *(Potri.003G118400)* | GCCTTGCCTGAGATACTTAC | GCTTCAGTCATAGTCTTCAC |
| *SOD2 (Potri.009G005100)* | CTAATGTTGAAGGCGTCGTC | ACGCATCCATTTGTTGTGTC |
| *SOD3 (Potri.006G049100)* | CTCTCATTCTCCTCTCCGTG | CCAGGCACACCACCAACTTG |
| *POD1 (Potri.016G084800)* | GTCTTATGCTGACTTCTACC | AATCCAGAACGCTCCTTGTG |
| *POD2 (Potri.016G132700)* | CAGGCTGCTTTCAGGACAGACTTTG | GATCTGGCCATTTGTGCCAGTAAGTG |
| *POD3 (Potri.007G053400)* | GACTCCAGAATAGCCATCAACATGG | GGCTTGTTGAAAGGCCTGTGAGTC |
| *P5CS1 (Potri.010G198400)* | AGCGTCTCATTATCAAGGTTGGGAC | CAGCACCAGATGTCACCACAATAAC |
| *APX1 (Potri.009G015400)* | GCTCCTCTCATGCTTCGTCT | TCTGCCGAGTACCTCATGGT |
| *APX2 (Potri.006G132200)* | GCTTGCCTGATGCAACCAAA | TTGTGAACAGACCAGAGTGTG |
| *Actin (JM986590)* | ACCCTCCAATCCAGACACTG | TTGCTGACCGTATGAGCAAG |
| *EF1 (FN356200)* | AAGCCATGGGATGATGAGAC | ACTGGAGCCAATTTTGATGC |
