# Supplementary material for: PagERF16 of Populus Promotes Lateral Root Proliferation and Sensitizes to Salt Stress
Source: Front Plant Sci. 2021 Jun 4;12:669143. doi: 10.3389/fpls.2021.669143 (PMC8213033; doi:10.3389/fpls.2021.669143)
Supplement: Supplementary Table 2 — Statistic analysis of leaf color of OX, RNAi, and WT using LAB methods. 0 < ΔL, white; 0 > ΔL, black; 0 < ΔA, red; 0 > ΔA, green; 0 < ΔB, yellow; 0 > ΔB, blue. ΔE, color difference, ΔE* = [(ΔL*)2 + (ΔA*)2 + (ΔB*)2]1/2,1 < ΔE ≤ 2, not significant; 2 < ΔE, the difference is visible. [file Table_2.docx]

|  | Genotype | L | A | B | E |
| --- | --- | --- | --- | --- | --- |
| 0 mM | WT | \| 30.10 ± 3.803 \| \| --- \| | \| -12.90 ± 2.189 \| \| --- \| \|  \| | \| 21.86 ± 4.480 \| \| --- \| \|  \| |  |
|  | OX | \| 37.36 ± 4.551 \| \| --- \| \|  \| | \| -16.72 ± 1.449 \| \| --- \| \|  \| | \| 38.12 ± 7.042 \| \| --- \| \|  \| |  |
|  | Δ | 7.26 | -3.82 | 16.26 | 18.21 |
|  | RNAi | \| 31.08 ± 3.874 \| \| --- \| \|  \| | \| -15.18 ± 1.546 \| \| --- \| \|  \| | \| 29.57 ± 5.542 \| \| --- \| \|  \| |  |
|  | Δ | 0.98 | -2.28 | 7.71 | 8.09 |
| 50 mM | WT | \| 28.79 ± 2.938 \| \| --- \| \|  \| | \| -13.99 ± 1.756 \| \| --- \| \|  \| | \| 23.01 ± 4.648 \| \| --- \| \|  \| |  |
|  | OX | \| 31.44 ± 3.734 \| \| --- \| \|  \| | \| -14.28 ± 1.586 \| \| --- \| \|  \| | \| 26.05 ± 4.678 \| \| --- \| \|  \| |  |
|  | Δ | 2.65 | -0.29 | 3.04 | 4.043 |
|  | RNAi | \| 31.01 ± 3.502 \| \| --- \| \|  \| | \| -14.21 ± 1.585 \| \| --- \| \|  \| | \| 25.12 ± 3.853 \| \| --- \| \|  \| |  |
|  | Δ | 2.22 | -0.22 | 2.11 | 3.07 |

Table S2 Statistic analysis of leaf color of OX, RNAi and WT plants using LAB methods.

ΔE, Color difference, ΔE* = [(ΔL*)^2^+(ΔA*)^2^+(ΔB*)^2^]^1/2^,1 < ΔE ≤ 2, not significant; 2 < ΔE, the difference is visible. 0 < ΔL, white; 0 > ΔL, black; 0 < ΔA, red; 0 > ΔA, green; 0 < ΔB, yellow; 0>ΔB, blue.
